# Norman Rockwell (1894-1978). Postman Reading Mail (Saturday Evening Post cover, 18 February 1922).

**DOI:** 10.3201/eid0810.02-1000

**Published:** 2002-10

**Authors:** Polyxeni Potter

**Affiliations:** * Centers for Disease Control and Prevention, Atlanta, Georgia, USA

**Figure:**
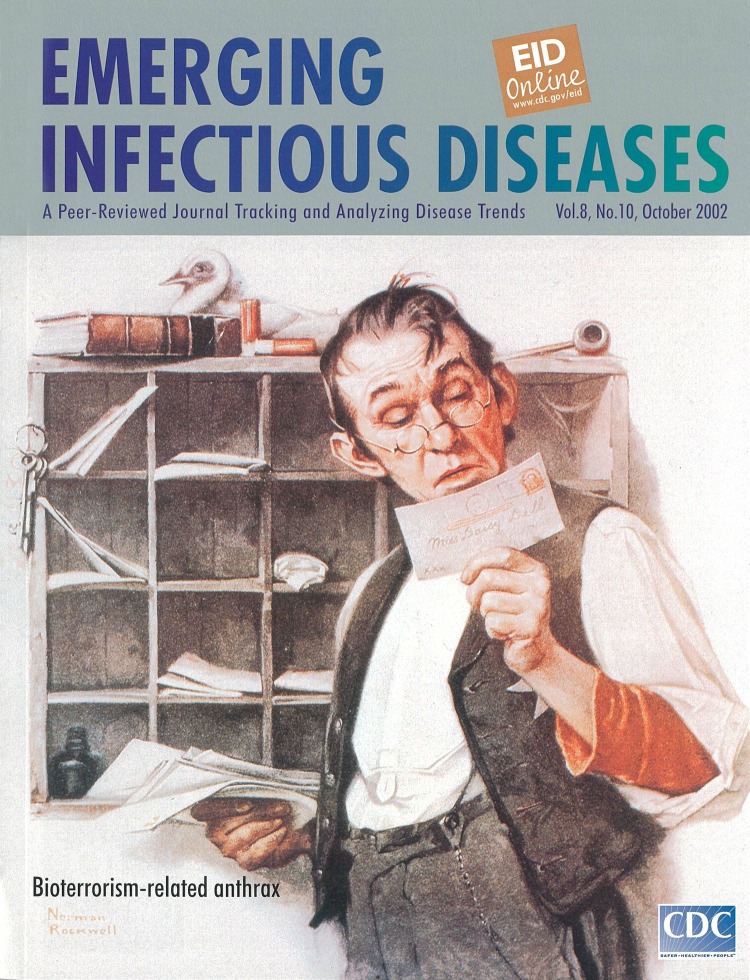
**Norman Rockwell (1894-1978). Postman Reading Mail (Saturday Evening Post cover, 18 February 1922).** Courtesy of the Curtis Publishing Company, Indianapolis, Indiana, USA.

Norman Rockwell, North America's most beloved and certainly best known illustrator, favored scenes of everyday life and reveled in his ability to tell stories. His Dickensian view of life drove him to paint the world as he would like it to be—no drunken fathers or self-centered mothers, only kindly doctors, duty-bound soldiers, and regular folks at their daily occupations. In his pictures, the sadness was wistful and the problems humorous. Rockwell, who presented himself as an illustrator rather than a fine arts painter, was also an interpreter of the classics and a recorder of history and the contemporary scene, from the Nuclear Test Ban Treaty to the Civil Rights Movement (1).

The realism in Rockwell's illustrations was not photographic. Along with the artful detail, his cast of characters (teachers, students, models, homemakers) was loaded with nuances bestowed by the illustrator's genius. The characters sparkled, glowed, and communicated directly with the audience. And the message they sent was exactly the one Rockwell intended the audience to receive. Very much in touch with his surroundings, the artist lived many of the situations that eventually became the subjects of his pictures. He painted what he knew to be the life of a child, a student, a soldier, or a workingman, whether in the Mamaroneck, New York, of his youth, or anywhere else he lived after that. As a result, his characters were universal and accessible to the average viewer.

Rockwell's brief experience with the U.S. Postal Service, which may have colored his many depictions of postal workers of all ages, was in the eighth grade. To raise money for art school tuition, he bought the mail route to exclusive Orienta Point from another boy for $25. The wealthy residents of the Point each paid the mail carrier 25 cents to deliver the mail because the regular carrier did not deliver that far from town. Every morning at 5:30, rain or shine, Norman bicycled to the post office, loaded the mail into a leather shoulder bag, and rode 2.5 miles to the end of the Point, delivering the mail to homes on the way.

When "Postman Reading Mail" first hit the stands on the cover of the Saturday Evening Post, thousands of letters from postal workers protested the nosy behavior ascribed to one of their own. Rockwell fielded the protests graciously, explaining that the post office was a small operation, in a tiny town, with a few boxes of mail to sort, and the postal clerk had succumbed to boredom and human curiosity: "…if you are interested in the characters that you draw, and understand them and love them, why the person who sees your picture is bound to feel the same way." (Curtis Publishing Co. comm., 2002).

 If premier illustrator Norman Rockwell were alive today, he would be painting a different mail scene from the one featured on the cover of the Saturday Evening Post in 1922 and now on the cover of Emerging Infectious Diseases. Under the current circumstances of the world, in which the routine and harmless activity of sorting and delivering the mail was deliberately contaminated with a dreaded disease, this fine recorder of history would probably forego the humor in the scene.
